# Neuronal coding in the rodent prefrontal cortex

**DOI:** 10.1186/1471-2202-14-S1-P117

**Published:** 2013-07-08

**Authors:** Olga Kornienko, Liya Ma, James M Hyman, Jeremy K Seamans, Daniel Durstewitz

**Affiliations:** 1 Bernstein Center for Computational Neuroscience, Central Institute of Mental Health, Medical Faculty Mannheim, Heidelberg University, 68159, Germany; 2 Brain Research Centre, Psychiatry, Faculty of Medicine, University of British Columbia, Vancouver, BC, BC V6T 2B5, Canada

## 

Apart from being associated with working memory, neurons in the rodent medial prefrontal cortex (mPFC) are known to be involved in encoding of spatial and temporal contexts [1], the deduction of rules [2], and decision making [3]. The context-dependent organization of neural assemblies encoding for different task events, stimuli or decisions [3,4], may account for the great flexibility required during the performance of higher cognitive tasks. The way in which single neurons and their interactions code for different entities may play a huge role in this flexibility [5], but has rarely been systematically investigated in the PFC.

Here, we employ various multivariate statistical techniques and time series bootstraps to analyze the way in which neurons, neural interactions, and temporal patterns of activity within ensembles of simultaneously recorded rat PFC neurons contribute to the neural population code during the performance of different tasks comprised of multiple stimuli, task events, and responses.

To examine the neural population representation of a given set of stimuli and task events, in a first step kernel density stimates of spiking activity were obtained from all recorded neurons. Both multivariate/ multiple regression and classification approaches were then utilized to characterize neuronal coding properties. Using regression, the distributions of single neuron contributions to the explained variation in stimulus conditions were charted, both individually and after regressing out or taking into account the contribution of other neurons. The same was done including neuronal interaction terms of various orders as well as time-lagged versions of the neuronal activities (based on the idea of delay-embedding, thus taking temporal patterns into account). Significant contributions of single terms or sets of terms were identified by construction of null hypothesis distributions through block-permutation bootstraps. In a complementary decoding-type of analysis, a linear discriminant analysis (LDA) classifier was run on sets of single neuron activities, time-lagged versions of these, and their interaction terms, with performance evaluated through leave-one-out cross-validation. Results show that 1) contributions to explained variation in stimulus conditions follow monotonically falling, potentially power-law-like, distributions, and 2) both including temporal pattern information as well as neural interaction terms significantly improves prediction performance and strongly reduces the misclassification rate.

These findings indicate that a) there appears to be no highly specialized subpopulation of neurons encoding for specific events, and b) that precise temporal patterns, and to a lesser degree correlations among units, have a major contribution to the neural representation of specific stimuli and internal task stages in the rat mPFC.
